# White matter variations in congenital adrenal hyperplasia: possible implications for glucocorticoid treatment

**DOI:** 10.1093/braincomms/fcae334

**Published:** 2024-09-26

**Authors:** Eileen Luders, Debra Spencer, Christian Gaser, Ajay Thankamony, Ieuan A Hughes, Umasuthan Srirangalingam, Helena Gleeson, Karson T F Kung, Ryan P Cabeen, Melissa Hines, Florian Kurth

**Affiliations:** Department of Women’s and Children’s Health, Uppsala University, Uppsala SE-751 05, Sweden; Swedish Collegium for Advanced Study (SCAS), Uppsala 75238, Sweden; School of Psychology, University of Auckland, Auckland 1142, New Zealand; Laboratory of Neuro Imaging, USC Stevens Neuroimaging and Informatics Institute, Keck School of Medicine of USC, University of Southern California, Los Angeles, CA 90033, USA; Department of Psychology, University of Cambridge, Cambridge CB2 1TN, UK; Department of Psychiatry and Psychotherapy, Jena University Hospital, Jena 07747, Germany; Department of Neurology, Jena University Hospital, Jena 07747, Germany; Department of Paediatrics, Addenbrooke’s Hospital, University of Cambridge, Cambridge CB2 0QQ, UK; The Weston Centre for Paediatric Endocrinology and Diabetes, Addenbrooke’s Hospital, University of Cambridge, Cambridge CB2 0QQ, UK; Department of Paediatrics, Addenbrooke’s Hospital, University of Cambridge, Cambridge CB2 0QQ, UK; Department of Endocrinology and Diabetes, University College Hospital London, London NW1 2BU, UK; Queen Elizabeth Hospital, Birmingham B15 2GW, UK; Department of Psychology, The University of Hong Kong, Hong Kong 999077, China; Laboratory of Neuro Imaging, USC Stevens Neuroimaging and Informatics Institute, Keck School of Medicine of USC, University of Southern California, Los Angeles, CA 90033, USA; Department of Psychology, University of Cambridge, Cambridge CB2 1TN, UK; School of Psychology, University of Auckland, Auckland 1142, New Zealand; Department of Diagnostic and Interventional Radiology, Jena University Hospital, Jena 07747, Germany

**Keywords:** androgens, brain, corticosteroid, MRI, hyperintensities

## Abstract

Congenital adrenal hyperplasia has been reported to manifest with white matter aberrations. However, many previous studies included only small samples, restricted their analyses to females, lacked a control group and/or did not correct for brain size. Here, we examined the largest sample to date, comprising 53 male and female participants with congenital adrenal hyperplasia, who were matched with 53 male and female controls in terms of sex, age, education, and verbal intelligence. The four groups were compared with respect to their total white matter as well as white matter hyperintensities while applying brain size corrections. For both measures, total white matter and white matter hyperintensities, there were no significant sex differences or group-by-sex interactions. However, individuals with congenital adrenal hyperplasia had significantly smaller total white matter volumes compared to controls. Our findings align with previous reports of white matter variations in congenital adrenal hyperplasia. The absence of a group-by-sex interaction suggests that white matter variations in congenital adrenal hyperplasia may not be attributable to prenatal androgens. Instead, they may be a result of the condition itself and/or its treatment with glucocorticoids. The latter aspect warrants follow-up, particularly given that glucocorticoids are employed not only in congenital adrenal hyperplasia but also in other medical conditions.

## Teaser

Luders *et al*. detected less white matter in individuals with congenital adrenal hyperplasia compared to matched controls. Diminished white matter could be a result of the condition itself and/or of its treatment with glucocorticoids.

## Graphical Abstract

**Graphical Abstract:**
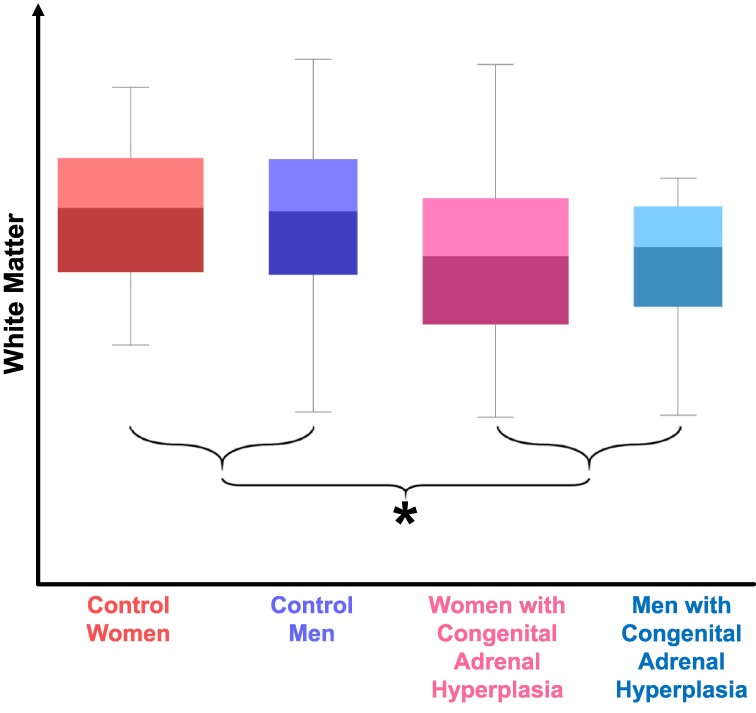

## Introduction

Congenital adrenal hyperplasia (CAH) refers to a group of genetic disorders which affect the adrenal glands. Among other things, CAH is accompanied by altered concentrations of cortisol and androgens,^1^ which are commonly treated with exogenous glucocorticoids. However, this treatment may result in overly elevated or abnormal levels of glucocorticoids which, in turn, may interfere with neuronal maturation (including myelination) and also exacerbate neurotoxic insults.^2^ Thus, glucocorticoid replacement therapy may have a negative impact on the brain by changing its underlying microstructure (e.g. demyelinated axons) and resulting macrostructure (e.g. smaller white matter volumes). Indeed, as recently reviewed,^1,3^ individuals with CAH can present with white matter abnormalities, such as white matter hyperintensities, white matter lesions, or reduced fractional anisotropy (an indicator of white matter microstructural integrity).

Nevertheless, the number of existing studies is limited and sample sizes are small, perhaps because CAH, and especially classic CAH, is rare with an incidence of ∼1 in 10 000–20 000.^4^ Aside from several case studies (*N* = 1) reviewed elsewhere,^1^ there are only eight studies examining white matter in CAH. These studies range with respect to the number of individuals with CAH from *N* = 7 (+3 controls),^5^ to *N* = 19 (+19 controls),^6,7^ to *N* = 22 (no controls),^8^ to *N* = 23 (+33 controls),^9^ to *N* = 26 (no controls),^10^ to *N* = 39 (no controls)^2^ and to *N* = 43 (+43 controls).^11^ Small sample sizes decrease statistical power, and while low statistical power diminishes the chance of detecting a true effect (e.g. the study with an *N* of 7 reported a lack of white matter abnormalities), it also reduces the likelihood that a statistically significant result reflects a true effect.^12^ Moreover, not all studies have accounted for the typically larger brain size in males compared to females, and those that did sometimes found that the initially observed white matter effects were no longer significant.^11^ Furthermore, some studies did not include a control group^2,8,10^ and some only included females with CAH but not males with CAH.^6^

Clearly, more research is necessary to understand possible white matter abnormalities in CAH. Thus, here, we analysed the largest sample to date comprising 53 individuals with CAH and 53 well-matched controls; we included both women (33 CAH/33 controls) and men (20 CAH/20 controls) and also applied appropriate corrections for brain size.

## Materials and methods

### Study sample

The present study is part of an NIH-funded project, entitled ‘Brain and behavior in individuals with intersex conditions’ (R01HD081720). The study was based on 53 individuals with classic CAH^4,13^ who were matched pair-wise to 53 controls with respect to sex, age, education, and verbal intelligence. Sample characteristics are summarized in Table 1; for additional details, refer to prior publications.^15,16^ Approval for the study was obtained from an NHS Research Ethics Committee and the Health Research Authority in the UK (15/EM/0532) as well as the Ethics Committee at the University of Auckland in New Zealand (020825). All participants provided their informed consent.

**Table 1:** Sample characteristics

	Control women	Control men	Women with CAH	Men with CAH
*N*	33	20	33	20
Age in years				
Mean ± SD	31.8 ± 8.5	27.9 ± 5.5	31.1 ± 8.6	28.5 ± 6.6
Range	18.3–45.3	19.4–40.8	18.3–45.7	19.3–43.4
Verbal intelligence^a^				
Mean ± SD	6.3 ± 2.3	6.4 ± 3.1	6.3 ± 2.6	5.6 ± 3.4
Range	1.8–11.0	−1.0 to 13.5	1.5–11.2	2.0–12.5
Education^b^				
Mean ± SD	4.1 ± 1.3	3.9 ± 1.2	4.0 ± 1.3	3.8 ± 1.4
• GCSEs	*n* = 6	*n* = 3	*n* = 6	*n* = 4
• A Levels	*n* = 6	*n* = 5	*n* = 5	*n* = 7
• Vocational training	*n* = 5	*n* = 4	*n* = 6	*n* = 1
• Bachelor’s degree	*n* = 12	*n* = 7	*n* = 14	*n* = 5
• Master’s degree	*n* = 4	*n* = 1	*n* = 2	*n* = 3

SD, standard deviation; GCSE , General Certificate of Secondary Education.

^a^Measured using the advanced vocabulary test^14^.

^b^Highest level of education obtained, coded as GCSEs = 2; A levels = 3; vocational training = 4; bachelor’s degree = 5; master’s degree = 6.

### Brain image acquisition

All participants were scanned between 2016 and 2022 on the same Siemens 3.0 Tesla Skyra system, as detailed elsewhere.^15^ The collected T_1_-weighted structural images underwent quality control using visual inspections as well as objective criteria implemented in CAT12.^17^ Of the originally acquired 110 brain images, two brain images from the CAH group did not pass and as such had to be removed. In order to retain the well-matched sample, the corresponding two brains of the control group were removed as well, which resulted in a final sample of 53 individuals with CAH and 53 controls.

### Brain image analyses

All images were processed using CAT12,^17^ applying inhomogeneity corrections and tissue classifications, as previously detailed.^18,19^ The total white matter volume was calculated in millilitres (mL) by adding up the partial volumes of the voxels classified as containing white matter. In order to derive the individual estimates for the white matter hyperintensities, we used the automated procedure implemented in CAT12, which included an optimized low-resolution shooting registration technique^20^ as well as a fine-grained local correction based on region-growing and bottle-neck algorithms.^21,22^ The output is an individual brain map of white matter hyperintensities (see Supplemental Fig. 1), which are any isolated clusters of pathological features within the white matter as well as any voxels surrounding the lateral ventricles that present with an intensity typical for grey matter but have a high probability for white matter. The volume of the white matter hyperintensities was calculated (in mL) by adding up the volumes of the respective voxels.

### Statistical analyses

Statistical analyses were performed in MATLAB (https://www.mathworks.com/products/matlab.html) using general linear models. The total white matter volumes and white matter hyperintensity volumes were entered as the *dependent* variables; group (CAH/control), sex (female/male), and the group-by-sex interaction as the *independent* variables; and age and total intracranial volume (TIV)^16^ as *variables of no interest*. Significance was established using Monte Carlo simulations with 10 000 permutations to avoid relying on assumptions for parametric testing. Results were corrected for multiple comparisons resulting from the two dependent variables by controlling the family-wise error (FWE) rate.^23^ TIV, as well as *raw* volumes and *adjusted* (corrected for age and TIV) volumes of total white matter and white matter hyperintensities are provided in Table 2.

**Table 2:** Total intracranial, raw white matter, and adjusted white matter volumes (mean ± SD)

	Control women	Control men	Women with CAH	Men with CAH
Total intracranial volume in mL				
TIV	1399.1 ± 99.1	1585.5 ± 118.7	1361.5 ± 117.8	1549.1 ± 154.0
Raw white matter volumes in mL				
Total white matter	494.6 ± 42.7	561.8 ± 55.3	468.7 ± 50.8	536.9 ± 62.9
White matter hyperintensities	0.8 ± 0.5	0.8 ± 0.4	0.8 ± 1.2	0.8 ± 0.3
Adjusted white matter volumes (corrected for age and TIV) in mL				
Total white matter	512.7 ± 27.2	512.5 ± 24.7	502.3 ± 26.3	500.9 ± 18.1
White matter hyperintensities	0.8 ± 0.5	0.7 ± 0.4	0.8 ± 1.2	0.7 ± 0.3

SD, standard deviation; TIV, total intracranial volume; mL, millilitres.

## Results

**Figure 1:**
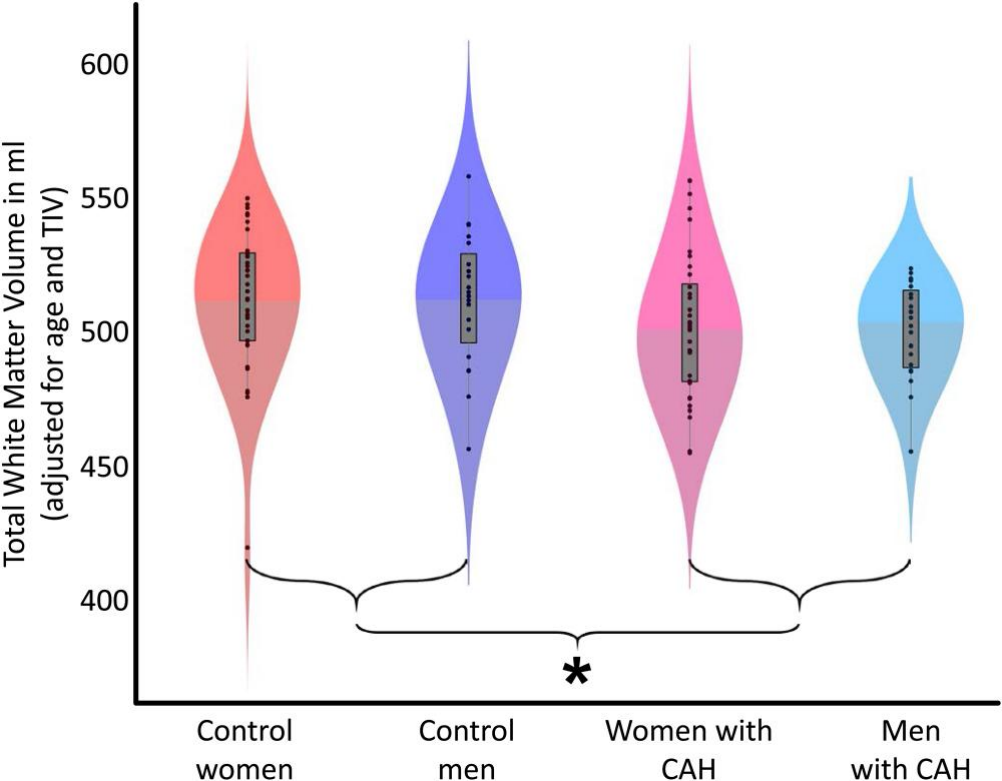
**Group-specific total white matter volumes.** The violin plots depict the distribution for each of the four groups. The black dots show individual volume estimates, the grey boxes show the group-specific interquartile ranges and the whiskers show the group-specific 1.5 interquartile ranges. The difference in shading indicates the median. Total white matter volumes are adjusted for age and TIV. The asterisk indicates the significant group difference (*P* = 0.045), with larger total white matter volumes in controls (33 women/20 men) than in individuals with CAH (33 women/20 men).

For total white matter, there was no significant main effect of sex (women versus men) and no significant group-by-sex interaction. In contrast, there was a significant main effect of group (CAH versus controls), with significantly smaller volumes in individuals with CAH (women + men) compared to controls (women + men). The group-specific distribution of the total white matter volumes adjusted for TIV and age (as per the statistical model) are depicted in Fig. 1. For white matter hyperintensities, there was no significant main effect of sex, no significant main effect of group, and no significant group-by-sex interaction. The statistics for total white matter as well as white matter hyperintensities are provided in Table 3.

**Table 3:** Results

Total white matter	F (df, df)	Significance (*P*)
Main effect of group	4.62(1100)	0.045^a^
Main effect of sex	0.02 (1100)	0.993
Group-by-sex interaction	0.01 (1100)	0.994

White matter hyperintensities	F (df, df)	Significance (*P*)
Main effect of group	0.04 (1100)	0.987
Main effect of sex	0.37 (1100)	0.842
Group-by-sex interaction	0.07 (1100)	0.970

^a^Significant at *P* ≤ 0.05, corrected for multiple comparisons.

## Discussion

To our knowledge, this is the largest study to date (*N* = 106) examining white matter in a well-matched sample of men and women with and without CAH. With respect to the observed aberrations in total white matter, the present findings are in line with prior reports of white matter abnormalities in CAH in general.^2,6,8,10,11,24-28^ The current absence of significant group differences with respect to white matter hyperintensities seems to conflict with some prior reports.^7,8,10,24-26^ However, some of those studies only consisted of a single patient,^24-26^ lacked a control group^8,10^ and/or only reported hyperintensities in a small fraction of patients without actually applying any statistical tests.^7,8,10^ Moreover, the majority of studies that revealed significant effects in regards to white matter hyperintensities did so using T_2_-weighted images, FLAIR images, or diffusion-weighted images, rather than T_1_-weighted images, as done in the current study. In fact, some of the studies that included both T_1_-weighted and otherwise weighted images reported an absence of significant effects specifically when using the T_1_-weighted images.^24,28,29^ Thus, future research may benefit from combining measures based on multiple magnetic resonance imaging modalities (e.g. T_1_-weighted, T_2_-weighted, FLAIR, and diffusion tensor imaging), while including appropriate control groups and testing for statistical significance.

Classic CAH causes elevated androgen exposure of female foetuses, whereas androgen levels in male foetuses are largely unchanged. Therefore, if foetal androgens affected white matter, one would observe a significant difference when comparing females with and without CAH (i.e. groups with different foetal androgen levels), but not when comparing males with and without CAH (i.e. groups with similar foetal androgen levels). Consequently, there would be a group-by-sex interaction. The absence of such an interaction, combined with the observed group effect (less total white matter in males and females with CAH compared to male and female controls) seems to suggest that white matter may be affected by aspects of the condition itself and/or by its treatment. More specifically, CAH is managed by administering exogenous glucocorticoids which mitigates cortisol deficiency and suppresses excess androgen production. This can result in an excessive glucocorticoid exposure, which may amplify neurotoxic insults and/or disrupt neuronal myelination.^2^ A limitation of our study is the absence of information on doses of glucocorticoids prescribed over time. However, as previously reported in an independent CAH sample,^6^ patients who were exposed to higher glucocorticoid doses had greater abnormalities in white matter microstructure (and also cognitive performance). Similar aberrant white matter has also been observed in other conditions where the administration of glucocorticoids is the primary treatment, such as in Duchenne muscular dystrophy (reviewed elsewhere^30^).

In summary, our findings align with previous reports of white matter abnormalities in CAH. The smaller white matter volumes in both men and women with CAH may indicate adverse treatment effects of glucocorticoids. This possibility carries substantial implications given the widespread use of glucocorticoids in the treatment of diverse medical conditions, including inflammatory, allergic and immunological disorders,^31^ as well as their vital role in supporting preterm neonatal lung development and overall health.^32^ Consequently, further research—ideally containing information on treatment regimen (especially glucocorticoid doses) as well as measures of cortisol and androgens—is required to understand the underlying mechanisms of these current findings and their implications for the management of CAH and other medical conditions.

## Supplementary Material

fcae334_Supplementary_Data

## Data Availability

The data are not publicly available due to ethical restrictions imposed by the signed consent. Any reasonable request for data access should be made to the corresponding author.
