# Supplementary material for: White matter variations in congenital adrenal hyperplasia: possible implications for glucocorticoid treatment
Source: Brain Commun. 2024 Sep 26;6(5):fcae334. doi: 10.1093/braincomms/fcae334 (PMC11467690; doi:10.1093/braincomms/fcae334)
Supplement: fcae334_Supplementary_Data [file fcae334_supplementary_data.pdf]

Supplemental Figure 1

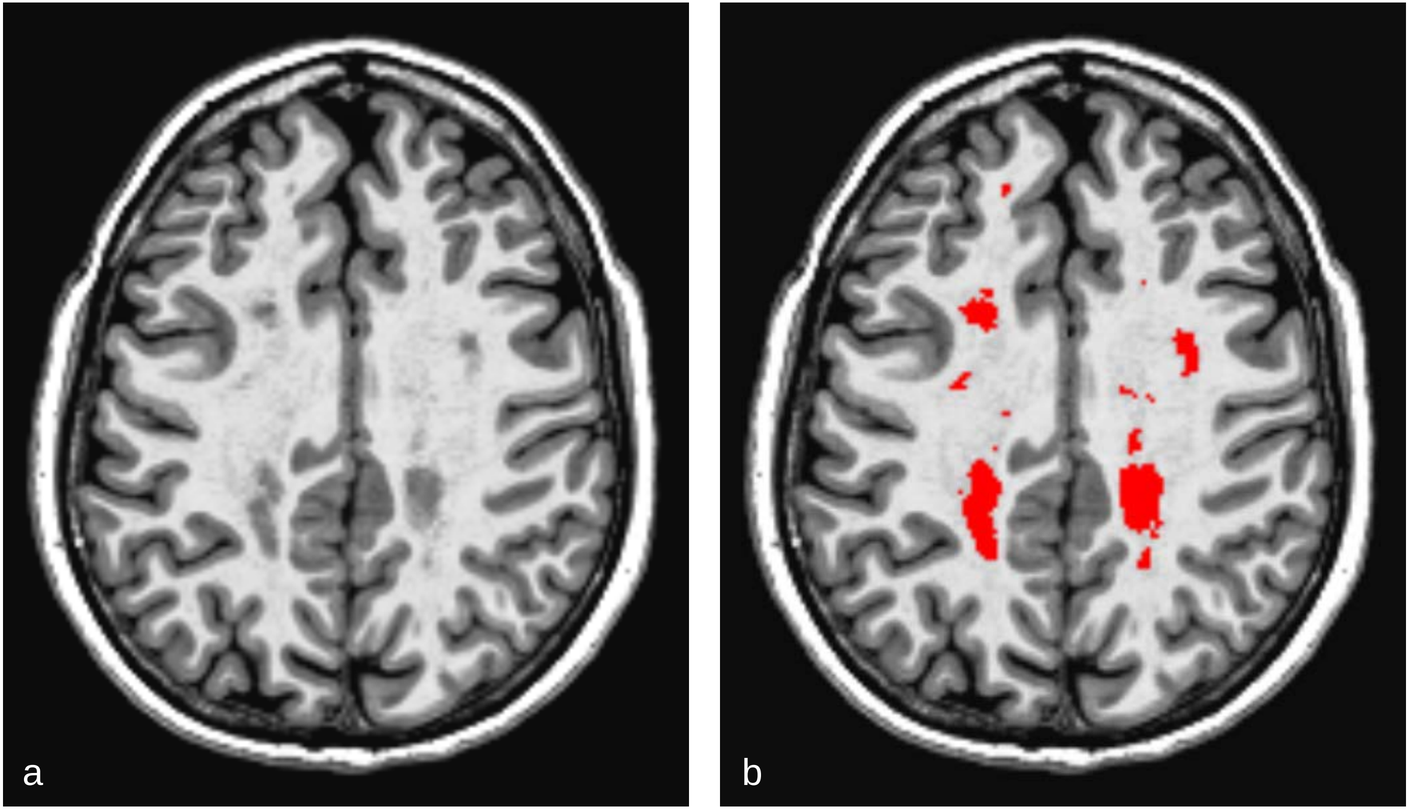

White matter hyperintensities (WMHs). Panel **a:** Axial section of a brain with a high load of WMHs. Panel **b**: The same axial section with the WMHs labeled in red.
